# Social participation challenges and facilitators among Chinese stroke survivors: a qualitative descriptive study

**DOI:** 10.1186/s12889-025-21592-z

**Published:** 2025-02-05

**Authors:** Xiaojuan Wan, Dorothy Ngo Sheung Chan, Janita Pak Chun Chau, Yu Zhang, Zhi’e Gu, Limei Xu

**Affiliations:** 1 School of Nursing, Yangzhou University, 136#, Hanjiang Middle Road, Yangzhou, Jiangsu Province Mainland China; 2 Nethersole School of Nursing, Faculty of Medicine, The Chinese University of Hong Kong, Esther Lee Building, Shatin, N.T., Hong Kong SAR China; 3 Northern Jiangsu People’s Hospital, Affiliated Hospital of Yangzhou University, 98#, Nantong West Road, Yangzhou, Jiangsu Province Mainland China; 4 Wenfeng Community Health Service Centre, No. 11 East Garden South of Henan Bridge, Yangzhou, Jiangsu Province Mainland China

**Keywords:** Challenges and facilitators, Cultural values, Qualitative study: social participation, Social support, Stroke survivors

## Abstract

**Background:**

A significant proportion of stroke survivors have participation restrictions. Attaining an in-depth understanding of participation challenges and facilitators is necessary to identify rehabilitation priorities. However, current evidence in the Chinese context is insufficient as cultural norms, expectations, and behaviors may differ across countries. This study aimed to explore experiences of social participation among Chinese stroke survivors and their perceived challenges and facilitators, particularly the cultural issues that impact their social participation.

**Methods:**

This study used a qualitative descriptive approach. Individual face-to-face semi-structured interviews were conducted with 30 first-time or recurrent stroke survivors recruited from a rehabilitation unit and a community health centre in China. Content analysis was used to analyse the data. Reporting complies with the COREQ checklist.

**Results:**

Three investigator-derived categories and 14 subcategories based on data-derived responses were identified. The categories included changes in social participation after stroke, challenges of social participation, and facilitators of social participation. While common factors such as physical limitations, environmental features, and social support emerged as influences on social participation, cultural values were also found to exert a significant impact on survivors’ participation behaviors. These values included family-oriented beliefs regarding responsibility towards their children, struggles with societal expectations, and perceptions of social participation being a burden on others.

**Conclusions:**

Stroke survivors perceived varying degrees of changes in their social participation. Specifically, this study identifies the influence of prevalent cultural values on survivors’ participation behaviors, indicating that cultural beliefs and the associated effects on health outcomes should be taken into consideration when developing interventions to enhance social participation after stroke. Potential interventions to empower survivors could aim to improve their stroke-related knowledge, provide psychological encouragement, incorporate skills training to improve communication with family members, and organise peer support groups to augment social support networks.

**Supplementary Information:**

The online version contains supplementary material available at 10.1186/s12889-025-21592-z.

## Introduction

Stroke is the second leading cause of death and third leading cause of disability-adjusted life-years lost globally [1]. Stroke survivors face substantial psychological and social challenges, such as emotional distress and difficulty in community reintegration [2–3]. A significant proportion of stroke survivors find returning to their previous levels of social participation challenging and many have to live with long-term participation restrictions which are associated with recurrent stroke, increased mortality, and lower quality of life [4]. Stroke survivors often identify restoring social participation to pre-stroke levels as a high-priority rehabilitation goal [5]. However, interventions that target the psychosocial outcomes of stroke survivors, especially with regard to their social participation outcomes post-stroke, are still lacking [6–7]. Thus gaining in-depth insights into stroke survivors’ experiences of social participation and understanding their challenges and facilitators is necessary to inform the development of relevant and targeted interventions.

There is a degree of complexity and ambiguity when it comes to conceptualizing social participation in research and practice. Researchers have argued that there is no clear distinction between “social participation” and “participation” [8–9]. According to a content analysis, the majority of definitions of social participation focused on the individual’s involvement in activities that allowed them to interact with others in society or the community [10]. This aligns with the “activity and participation” classification under the World Health Organization’s International Classification of Functioning, Disability and Health (ICF) [9]. As outlined by the ICF classification system, participation consists of four facets (Chap. 6 to Chap. 9): participation in domestic life; interpersonal interactions and relationships; major life areas (e.g., work, education, and economic life); and community, social, and civic life [9, 11].

Existing qualitative literature suggests that stroke survivors’ experience of social participation is influenced by a number of factors, including physical impairment, psychological health, social support, and physical environment [12–14]. However, less attention has been paid to the influence of cultural values on survivors’ participation [9]. Particularly as cultural beliefs, norms, and expectations can influence health behaviours [15], there may be differences in participation experiences across countries and regions [16]. Accordingly, the findings of most studies which are conducted in Western contexts may lack sufficient evidence to guide the development of interventions for stroke survivors in China [3].

A limited number of qualitative studies were found to have explored social participation and its related barriers and facilitators among Chinese stroke survivors [17–18]. Moreover, due to unique features of their samples which consisted of younger stroke survivors and residents in rural areas, the findings may not be generalisable to older populations or those living in urban areas as there may be significant differences in socioeconomic status, available health facilities, and prevailing stroke management practices [19–21]. In addition, few studies have explored social participation systematically within a framework [2–3].

Aiming to explore the experiences of social participation among stroke survivors in China and understand the associated challenges and facilitators using a qualitative study design, this study explored participation restrictions using ICF constructs as the guiding framework and also investigated the effect of cultural values on social participation. The ICF is a widely adopted framework in rehabilitation that describes health and health-related states. Participation, as one of the core components, is an essential source for understanding the impact of impairments and disabilities on an individual’s life. The ICF emphasizes that health problems, impairments, and activity limitations do not necessarily lead to restrictions in participation. This highlights the importance of considering the broader context of social and environmental factors in evaluating participation. A thorough understanding of participation, especially in the context of specific cultural settings, is crucial for developing interventions that are tailored to enhance social participation. It is anticipated that the findings of this study could help develop culturally relevant interventions to enhance social participation among stroke survivors, particularly in Chinese communities.

## Methods

### Design

A qualitative descriptive study design was adopted utilizing in-depth face-to-face semi-structured interviews. The report of this study is presented according to the COnsolidated criteria for REporting Qualitative research (COREQ) guidelines [22].

### Settings

Participants were recruited by purposive sampling from a rehabilitation unit and a community health centre in Yangzhou, China.

### Participants and recruitment

The eligibility criteria were as follows. Inclusion criteria: (1) having a clinical diagnosis of first-time or recurrent ischaemic or hemorrhagic stroke; (2) age ≥ 18 years; and (3) being able to communicate meaningfully in Mandarin and provide informed consent. Exclusion criteria: (1) medically unstable; (2) having a diagnosis of mental illness; (3) having moderate or severe cognitive impairments or unable to provide informed consent (e.g., Mini-Mental State Examination score of ≤ 20); (4) having severe dysphasia (e.g., aphasia) and being unable to communicate meaningfully (e.g., Boston Diagnostic Aphasia Examination < level 3); and (5) fully dependent, or unable to reach the interview sites, even with assistance (e.g. Modified Rankin Scale score of ≥ 5).

To ensure diversity among participants, efforts were made during recruitment to achieve a balance and variety in participants’ gender, age, time since stroke, and level of disability. The sample size was determined based on the principle of data saturation; i.e., interviews with new participants continued until no new information (subcategories or categories) emerged [23]. The research team reviewed the analyses, findings, and quality of participant quotations to determine whether there were enough in-depth data addressing the research questions to conclude data collection. The point of data saturation was determined to have been reached after interviewing 27 stroke survivors, and three more participants were interviewed to corroborate the findings.

Potential participants were identified by reviewing their health records and by consulting physicians or nurses. Those who met the inclusion criteria were then contacted by the physicians or nurses and invited to participate in the study. The principal investigator explained the full study to potential participants face-to-face before they signed the written informed consent form.

### Data collection

A semi-structured interview guide comprising open-ended questions was used for interviewing each participant (See Table 1). Throughout the interview, open-ended and non-leading prompts were provided as needed. The interview guide was piloted on three stroke survivors and the questions covered the experiences, challenges, and facilitators of social participation after stroke; further follow-up questions were asked as needed. The interviewer was an experienced researcher with a postgraduate degree and had been trained in conducting interviews. She also had a shared cultural background with the participants (Mainland Chinese). The individual interviews were conducted in a family physician centre of the community health centre or in a small conference room in the rehabilitation unit. Both locations had small and quiet rooms and no one except the interviewer and interviewee were allowed to enter, which guaranteed the privacy and comfort of participants. In cases of mild dysphasia, the interviewer invited caregivers to participate in the interview with the survivors’ consent. To aid understanding, various communication strategies were used for those who preferred to participate alone. These strategies included emphasizing specific words to help them comprehend the questions. Interviews lasted 30–40 min and were audio-recorded with participants’ informed consent. Field notes were made during the interviews, with documentation of non-verbal language and conversation context.

**Table 1:** Semi-structured interview guide

Questions	
(1) What changes did stroke bring to your life in terms of your participation in life/social activities, compared to life before stroke? [prompt: activities at home, in the rehabilitation unit, in the community, relationship with family and friends, paid or unpaid work] (2) What are the challenges in your participation in life after stroke (prompt: the changes in social participation mentioned above)? (3) What are the facilitators of your participation in life after stroke (prompt: the changes in social participation mentioned above)?	

### Data analysis

Participants’ demographic and clinical characteristics were descriptively presented as frequencies (percentages) or means (standard deviation). Content analysis (both manifest and latent content analysis) was used to analyze the qualitative data. Content analysis was conducted following the steps of data preparation and management, analytical trail development, and data interpretation [24–25]. During the data preparation period, interview data, which were audio tape recordings, were transcribed verbatim by one researcher and checked for accuracy according to the audio tapes by another researcher. The text files were imported to NVivo (Version 12). During the analytical trail development period, the coders read the transcripts several times to become immersed into the data and develop an overall understanding of the content. The meaning units, which reflected social participation experiences, challenges or facilitators, were identified, abstracted and coded. Coded data were then organized, and the investigator-derived categories and the subcategories based on data-derived responses were determined. Both inductive and deductive reasoning were used during content analysis. The abstraction and condensation of social participation experiences were linked to the ICF conceptual framework of participation [11]. The coding, abstraction, and condensation were conducted by two researchers, including the interviewer. The accuracy and objectivity of the data were ensured through strategies including independent coding of the interviews by two researchers, a review of the coding by an expert in qualitative research, and discussions among the research team.

All of the investigator-derived categories, subcategories based on data-derived responses, and corresponding quotations were translated from Chinese into English by two bilingual specialists in stroke rehabilitation. Back-translation was applied to ensure accuracy.

### Strategies to ensure trustworthiness

Several strategies were used to ensure the trustworthiness of the study in terms of credibility, transferability, dependability and confirmability [26]. Triangulation through data sources (recruiting a variety of participants through purposive sampling) and member checking was used to guarantee credibility [27]. In terms of member checking, the investigator-derived categories, subcategories based on data-derived responses, interpretations, and conclusions drawn from the transcripts after data analysis were shared and confirmed with relevant participants who were interested in commenting on or assessing the research findings. A comprehensive description of the study settings, participants’ characteristics and their experiences of social participation after stroke was obtained, allowing for comparisons between different settings and contexts, to check transferability [28]. In the current study, all of the research steps, from the study design to data collection and analysis, were described in detail; this will allow future researchers to repeat the process and address dependability issues [27]. During data collection and analysis, the researchers maintained a reflexive standpoint to maintain neutrality and objectivity in order to ensure that the study’s results were drawn from data rather than the researchers’ assumptions and to establish confirmability. For example, after each interview, the interviewer reflected on whether her unconscious responses during the interviews had influenced the interviewee’ expression.

## Findings

### Participant characteristics

From May to June 2021, 158 stroke survivors were screened, and 34 eligible participants were subsequently approached. Four eligible participants declined to participate: two stroke survivors declined because of physical discomfort; one declined because he was unable to reach the interview location by himself due to physical limitations; and one declined because she had no interest in the study. Ultimately, 30 stroke survivors were included and interviewed in this.

study. The age of the participants ranged from 50 to 81 years old. Sixteen participants were men (53.3%). The survivors’ time since their last stroke ranged from 2 months to 13 years. The characteristics of the participants are presented in Table 2.

**Table 2:** Characteristics of the participants (*n* = 30)

Participant	Gender	Age	Educational level	Duration after stroke	Modified Rankin Scale score	Monthly income
1	Male	67	High school	63 months	1	3000–5000 CNY (425–725 USD)
2	Female	65	Primary school	11 months	3	3000–5000 CNY (425–725 USD)
3	Female	68	Primary school	19 months	3	≤ 3000 CNY (425 USD)
4	Female	72	None	23 months	3	≤ 3000 CNY (425 USD)
5	Male	62	None	132 months	4	≤ 3000 CNY (425 USD)
6	Female	70	Primary school	28 months	3	5000–10,000 CNY (725-1,450 USD)
7	Male	52	Secondary school	3 months	4	3000–5000 CNY (425–725 USD)
8	Male	64	High school	10 months	4	5000–10,000 CNY (725-1,450 USD)
9	Male	53	High school	4 months	4	5000–10,000 CNY (725-1,450 USD)
10	Male	50	Secondary school	32 months	2	3000–5000 CNY (425–725 USD)
11	Male	71	High school	49 months	2	5000–10,000 CNY (725-1,450 USD)
12	Female	55	Secondary school	3 months	3	3000–5000 CNY (425–725 USD)
13	Female	60	Primary school	3 months	4	≤ 3000 CNY (425 USD)
14	Male	79	Primary school	9 months	4	≤ 3000 CNY (425 USD)
15	Male	53	Secondary school	96 months	3	≤ 3000 CNY (425 USD)
16	Female	71	Primary school	11 months	3	3000–5000 CNY (425–725 USD)
17	Male	65	High school	2 months	4	3000–5000 CNY (425–725 USD)
18	Male	56	Secondary school	20 months	2	5000–10,000 CNY (725-1,450 USD)
19	Male	66	High school	6 months	3	3000–5000 CNY (425–725 USD)
20	Male	70	Undergraduate	72 months	0	≥ 10,000 CNY
21	Female	67	Secondary school	3 months	3	3000–5000 CNY (425–725 USD)
22	Female	81	Secondary school	5 months	4	5000–10,000 CNY (725-1,450 USD)
23	Female	69	High school	18 months	3	5000–10,000 CNY (725-1,450 USD)
24	Male	75	Secondary school	8 months	3	3000–5000 CNY (425–725 USD)
25	Female	68	Secondary school	22 months	1	≤ 3000 CNY (425 USD)
26	Female	67	None	5 months	4	≤ 3000 CNY (425 USD)
27	Male	69	None	4months	4	≤ 3000 CNY (425 USD)
28	Female	68	High school	5 months	4	3000–5000 CNY (425–725 USD)
29	Female	74	High school	4 months	4	5000–10,000 CNY (725-1,450 USD)
30	Male	73	Undergraduate	156 months	0	≥ 10,000 CNY

### Qualitative findings

**Fig. 1:**
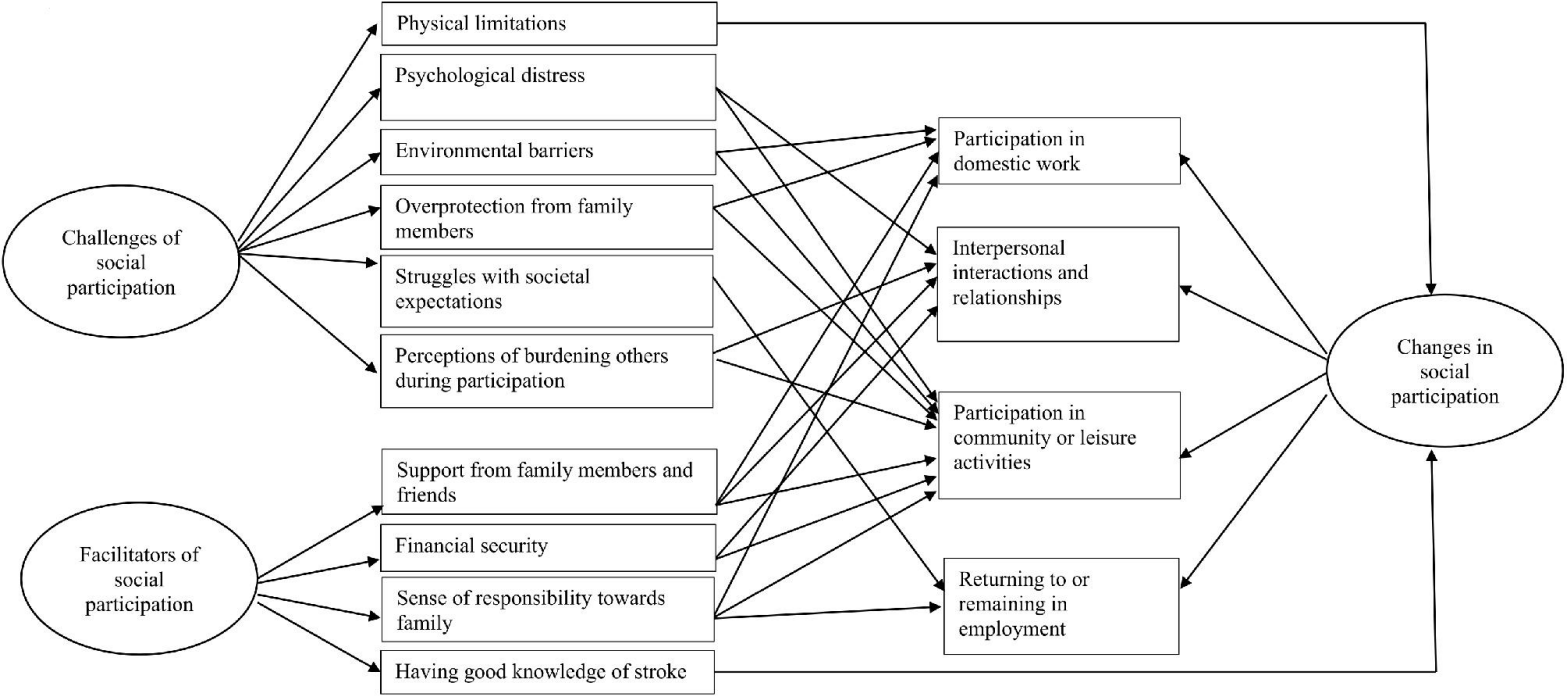
Factors contributing to the domains of social participation

Based on the investigators’ semi-structured interview guide, three investigator-derived categories were identified: changes in social participation after stroke, challenges of social participation, and facilitators of social participation. The investigator-derived categories, subcategories based on data-derived responses, and the ICF domains of participation impacted by facilitators/challenges are presented in Table 3. Figure 1 presents the various factors that contributed to the domains of social participation.

**Table 3:** Investigator-derived categories and subcategories based on data-derived responses

Categories (Investigator-derived categories)	Subcategories (Data-derived responses)
Changes in social participation after stroke	Decreased participation in domestic work
	Decreased interactions with friends and worsened relationships with family members
	Difficulty to return to or remain in employment
	Decreased participation in community or leisure activities
Challenges of social participation	Physical limitations
	Psychological distress
	Environmental barriers
	Overprotection from family members
	Struggles with societal expectations
	Perceptions of burdening others during participation
Facilitators of social participation	Having good knowledge of stroke
	Support from family members and friends
	Financial security
	Sense of responsibility towards family

### Changes in social participation after stroke

#### Decreased participation in domestic work

More than half of the stroke survivors reported decreased participation in domestic work such as cooking, cleaning, and looking after children, owing to their physical limitations after stroke. This change was mainly reported by older survivors who had retired and were mostly engaged in domestic work before stroke.

*“Prior to my stroke*,* I tackled all the household responsibilities*,* but now I can only manage lighter tasks now because of my hemiplegic leg.” (P16*,* 71 years old)*.

#### Decreased interactions with friends and worsened relationships with Family members

Many participants reported changes in their relationships with friends, relatives, and family members. Some reported that their interpersonal interactions with friends decreased due to their physical limitations or low motivation and others experienced strained relationships with their family members due to conflicts arising from the burdens of care or finances caused by their stroke.

“*My doctor has advised me to abstain from both alcohol and smoking. Consequently*,* I have chosen to refrain from attending gatherings with my friends since my stroke*,* as these events typically involve alcohol consumption and smoking*,* which I must now avoid.*” *(P11*,* 71 years old)*.

“*I quarrel with my wife almost every day. As I’m unable to stand up*,* I rely heavily on her assistance. As she can’t meet many of my needs*,* I become irritated and then furious. I find myself distressed*,* not knowing how to resolve this constant conflict. At times*,* in moments of desperation*,* I’ve resorted to slapping myself to ease the anger as I realize that striking her is not an option.*” *(P8*,* 64 years old)*.

#### Difficulty to return to or remain in employment

Due to various post-stroke limitations, such as hemiplegia or dysphasia, some participants who had been employed before their stroke quit their jobs, whereas others applied for early retirement or changed their occupation depending on their capacity after recovery. Job changes were mainly reported by younger stroke survivors who had been employed before their stroke.

“*Following my stroke*,* I had to quit my job and take on the role of a househusband.*” *(P10*,* 50 years old)*.

“*I was a taxi driver before stroke. I can’t drive anymore after my stroke*,* so I run a small shop now.*” *(P18*,* 56 years old)*.

#### Decreased participation in community or leisure activities

Many participants, especially those who had retired and participated in many community activities (e.g., dancing with friends in the community, playing cards, or playing chess) before the stroke, reported being involved in fewer community or leisure activities, primarily due to physical limitations.

*“Prior to my condition*,* I would routinely go out to dance with my friends. Sadly*,* my legs are now weakened and incapacitated*,* rendering me unable to dance as I once did. The only activity I am doing is walking slowly after meals.*” *(P2*,* 65 years old)*.

“*Before the stroke*,* I play cards with the neighbours every afternoon. But now one of my hands cannot hold the cards anymore. I have no leisure activities except watching TV.*” *(P8*,* 64 years old)*.

### Challenges of social participation

#### Physical limitations

Many participants reported physical limitations such as paralyzed or powerless limbs, decreased vision, fatigue, and pain, to be the central barriers to their participation. As the physical presence of survivors or their capacity to move independently was needed for most life situations, such as going outdoors for work, being able to safely do domestic chores, and maintain their social lives, this challenge impacted all domains of social participation.

“*I seldom go out because I cannot walk. My wife is too old to push the wheelchair for me.*” *(P14*,* 79 years old)*.

#### Psychological distress

Although stroke survivors could physically manage to participate in some activities after stroke, many of them reported that their social participation had greatly decreased because of poor psychological health, such as loss of interest, internalised stigma, loss of confidence, and fear of recurrent stroke. In particular, survivors noted that emotional distress resulting from stroke often hindered their willingness to engage with others and participate in community or leisure activities.

“*I do not want to be with other friends of my age who are running*,* dancing*,* and enjoying. I feel sad and ashamed compared with them because I cannot do anything now.*” *(P3*,* 68 years old)*.

#### Environmental barriers

Some participants reported that their physical environment was not suited to their participation needs, making it challenging for stroke survivors to engage in life situations. For example, some lived in a walk-up building, had unsafe home environments, or limited facilities for the disabled. These environmental barriers mainly impacted participation in domestic work and community or leisure activities.

“*I live on the 5th floor. There is no elevator. It is very difficult for me to go downstairs because of my hemiplegic limbs.*” *(P14*,* 79 years old)*.

“*Confined to a wheelchair*,* my mobility is limited to within the confines of my home. The challenge of boarding a bus in my current state is immense. You know… some sections of the street are also not suitable for wheelchairs.*” *(P8*,* 64 years old)*.

#### Overprotection from family members

After the stroke, family members normally took on more responsibilities and became increasingly protective of the participants. Since family members often considered it their duty to take care of the stroke survivors– reflecting the influence of family-oriented cultural values on caring for family members in need– they would express concern and discourage survivors from going outdoors or doing household chores which may put them in risky situations. However, some participants felt that this amounted to overprotection and became a barrier, which as a result, barred them from participating in domestic work and community or leisure activities.

“*Since the stroke*,* my daughter has not allowed me to travel to other places*,* even though I think I still can do so. She is afraid that an unforeseen accident may occur while I’m far from home and she is unable to provide timely assistance.*” *(P25*,* 68 years old)*.

“*My left limbs are powerless. My husband looks after me and he can nearly meet any of my needs. He does not allow me to do housework because he is afraid that I would fall. Since the stroke two years ago*,* I have not even tried to care for myself or go out alone.*” *(P4*,* 72 years old)*.

#### Struggles with societal expectations

The influence of societal expectations associated with the concept of filial piety also acted as a hindrance in survivors’ resumption of social participation. As it is expected that adult offspring will care for parents who are older or have limited working capacity, many older participants felt hesitant to resume or return to work as they believed that working for wages when they had physical limitations or after having retired would reflect poorly on their children and be considered socially unacceptable.

*“I’m doing some odd jobs to make money. I do not want others to know that I am still working for money after retirement and having had a stroke… the neighbours may laugh at me if they know this. They may think my children don’t support me.*” *(P1*,* 67 years old)*.

#### Perceptions of burdening others during participation

As stroke survivors often required help or accommodation from others during mobility, feelings of burdening others were reported as a barrier to social participation. Especially as survivors may require others to accommodate their needs such as accompany them while going outdoors, they perceived that their participation caused an imposition on others and was a significant burden to them. As a result, participants reported decreased interactions with friends and tried to participate in fewer activities in general so that they would reduce the burden of care on their families.

“*Since my stroke*,* I’ve been left with a leg that lacks strength*,* preventing me from walking swiftly. Consequently*,* I no longer join my friends for evening walks as I once did*,* as I’m aware they would have to slow down to match my pace. I don’t want to inconvenience them in such a way.*” *(P23*,* 69 years old)*.

“*I can’t walk long distances due to my powerless legs*,* so I have to rely heavily on my family for assistance when going out. I don’t want to bother my family frequently. Consequently*,* I now seldom leave the house*,* preferring not to burden my family with excessive demands*” *(P24*,* 75 years old)*.

Participants also thought that by going out they would burden fellow community members and thus, they did not go out often.

“*Sometimes I go out by myself in the wheelchair. However*,* there are times when I struggle to cross intersections within the duration of the green light*,* causing vehicles wait for me. When I cannot turn the wheelchair on uneven roads*,* some kind strangers have to help me. I don’t want to always bother others. I do not want to be a burden to the society*,* which is why I refrain from frequent outings.*” *(P26*,* 67 years old)*.

### Facilitators of social participation

#### Having good knowledge of stroke

Being well-informed about stroke was a facilitator of social participation as some participants were aware that engaging in life activities was beneficial to their brain health and could help them prevent recurrent strokes. This knowledge motivated them to actively participate in various activities across all domains of social participation.

“*I try to participate in all the activities that I can because I learnt that they are good for my brain health. Now*,* I am helping my friends revise articles before they submit them to the newspapers. I think doing this can help prevent stroke recurrence.*” *(P20*,* 70 years old)*.

“*Despite the discomfort and weakness in my leg*,* I persist in walking around the community three times daily because I know that my muscle strength will decrease if I stop walking.*” *(P19*,* 66 years old)*.

#### Support from family members and friends

Many participants reported that practical and emotional support from family members was a significant facilitator that helped them to reintegrate into society. Specifically, assistance from family members allowed survivors to resume participation in domestic work and in community and leisure activities. Some participants also remarked that support from friends helped them to integrate into their communities.

“*My legs aren’t as strong as before. My family took me anywhere I wanted*,* such as eating out*,* gathering with the family*,* and going to parks. I think I have adjusted very quickly after the stroke. I am very grateful to my family.*” *(P6*,* 70 years old)*.

“*I frequently go downstairs to chat with a bunch of friends who are also my neighbours. I find solace in their company when I’m feeling down*” *(P4*,* 72 years old)*.

#### Financial security

Several participants reported that having a stable income or retirement pension was a facilitator of social participation as certain activities, such as gathering with friends and eating outside, cost money. Notably, as older participants did not rely on their children for financial support, they felt more at ease to spend on socialising and leisure activities.

“*Maintaining contact with others invariably involves financial expenditure. If others give you some gifts or help you do something*,* you should give a gift in return to thank them. Consequently*,* if my retirement income were unstable*,* I would hesitate to frequently engage with friends.*” *(P6*,* 70 years old)*.

“*We enjoy the security of a stable retirement pension*,* which allows us (him and his wife) to engage in some money-requiring activities without placing any financial strain on our children. So*,* we often go out for some leisure activities*,* meeting some friends*,* or having morning tea.*” *(P20*,* 70 years old)*.

#### Sense of responsibility towards the family

Participants noted that they would try to participate in certain life aspects despite their disability because they felt a responsibility to do so. For domestic duties especially, survivors would try to provide childcare, complete chores, and visit relatives as they felt they owed it to their family members. A desire to contribute to their families financially through employment was also observed mostly in male participants. Such self-expectations of fulfilling personal roles and responsibilities in one’s family were facilitators of social participation.

“*As a housewife*,* doing housework is my responsibility. I must do it even though I do it very slowly now due to my hemiplegic limbs.*” *(P23*,* 69 years old)*.

“*In accordance with Chinese tradition*,* it is customary to visit relatives during festivals and exchange gifts as a gesture of affection. I must interact with relatives*,* especially during festivals*,* even though I do not want to meet them because of my bad body image.” (P24*,* 75 years old)*.

Many participants’ level of social participation was also dependent on their children’s living conditions as they were more willing to actively participate in social or leisure activities if their children had a comfortable life. In contrast, as they felt that parents have a responsibility to help their children for as long as their support is required, survivors were less likely to prioritise their participation needs if they felt their children still needed them.

“*Our children are leading fulfilling lives and are capable of navigating their own paths. As parents*,* we take pride in the fact that our responsibilities have been fulfilled. Therefore*,* despite the limitations I encounter following my stroke*,* we are living our own lives to the fullest*,* engaging in activities we can enjoy*,* such as playing cards and chess and so on.*” *(P11*,* 71 years old)*.

## Discussion

This study found that Chinese stroke survivors’ lives were impacted in every ICF participation domain and that their experiences were frequently influenced by prevalent cultural values. Notably, participants expressed significant concern regarding their limitations in participating in domestic work or fulfilling familial roles, which may differ from desired participation priorities observed in Western cultural contexts [29]. Specifically, we identified that certain cultural beliefs, such as those associated with the concept of filial piety, had a significant impact on social participation, indicating the necessity of developing culturally relevant interventions to improve survivors’ participation outcomes.

### Changes in social participation after stroke

Participants reported varying degrees of decreased social participation in domestic work, interpersonal interactions, work, and community or leisure activities. These changes in social participation have been observed in previous studies, with variations across groups with different demographic and cultural characteristics [30–31].

Survivors in the present study however were especially concerned about their participation restrictions in domestic chores and the fulfilment of familial roles, which is less frequently reported in studies conducted in Western countries. For example, a survey conducted in Sweden showed that the most frequently reported participation restrictions were in the domains of outdoor activities, work/education, social life and relationships. In contrast, participation restrictions in terms of fulfilling family responsibilities and domestic activities were less often reported [29]. This reflects disparities in reported participation restrictions across cultures, as different groups may value different activities and life roles depending on their cultural beliefs and associated expectations. Indeed, as the social unit of the “Family” is considered as the cornerstone of society in Chinese culture [32], Chinese stroke survivors prioritise their ability to take up family roles and duties, and frequently report concern resulting from a deterioration in their domestic life [31, 33], which was demonstrated in our study.

### Challenges and facilitators of social participation

Social participation was found to be influenced by physical and psychological states, knowledge of stroke, and social support, which is consistent across both Chinese and Western cultural contexts [17, 34].

Regarding psychological barriers, the issue of perceived stigmatisation was prevalent. Stroke survivors may experience a discrepancy in their social identity before and after stroke when reflecting on the loss of their former abilities or by interacting with others who lack an understanding of their condition. They may feel their social identity is stigmatised, leading to disruption or withdrawal from social relationships or interactions [35]. Consequently, survivors may miss out on opportunities to seek required health guidance as well as social support, which may further harm their recovery and participation outcomes [36]. It would therefore be helpful to introduce stroke survivors to accepting and supportive social networks, such as peer support groups, which may provide them with stroke-related guidance and advice in a welcoming context where they can de-stigmatise their condition and also gain the confidence to rebuild their personal and social identities through interaction with peers in similar situations [37].

### Influence of cultural values on social participation

Our study indicated that cultural values had a significant influence on stroke survivors’ social participation. Culture-related values that influence social participation included “Struggles with societal expectations” and “Perceptions of burdening others during participation”, which are subcategories of “challenges of social engagement”. The “Sense of responsibility towards family”, which falls under the category of “facilitators of social participation”, also influenced social participation.

#### Struggled with societal expectation

Arising from traditional Chinese beliefs on the importance of filial piety [38], many participants, especially those at an older age, believed that being employed after retirement is an indication that their children are unfilial since they cannot fulfil their needs, which is viewed as a shameful reflection on their family. Thus, if they chose to work after stroke, it may reflect poorly on their children’s ability to support them and would be considered a cultural taboo. This belief was a major barrier that prevented participants, especially those who had already retired, from returning to work or participating in relevant vocational training activities. To address this belief, communicating the positive health implications of social participation for survivors’ long-term health during interventions could provide survivors with an alternative perspective on the meaning and value of working after stroke. Moreover, emphasis can be placed on knowledge that engagement in specific activities, such as interpersonal interactions or social activities, can help to prevent cognitive decline and support occupational performance [39].

#### Sense of responsibility towards family

We also found that participants were more likely to have a higher level of social participation if they perceived that their children were settled in life and did not need their help. Given the family-centred values in Chinese culture [40], many parents believe in supporting their children even after they get married for as long as they need them (such as by taking care of grandchildren or doing housework) [41]. By contrast, if their children are already well-settled and cared for, parents feel that they have fulfilled their family responsibilities and can then concentrate on their own lives and actively participate in their personal and social lives [42]. As such, for those who feel hesitant to participate in social activities because of perceived family responsibilities, it is necessary to simultaneously support them in areas that they prioritise, such as by providing them with guidance on home modifications or functional rehabilitation exercises, as well as provide encouragement to resume engagement in other domains of social participation to improve their comprehensive recovery.

On the other hand, the sense of family responsibility also acted as a facilitator of participation in certain activities. As demonstrating persistence and responsibility is highly valued in Chinese culture [43], self-expectations regarding fulfilling personal roles and responsibilities, especially in family settings, motivated survivors to participate in life situations. It is a common cultural expectation among older people particularly that women are responsible for domestic work and men are responsible for earning a living [44], which may result in feelings of shame if they cannot fulfil their socially-prescribed responsibilities because of personal illness such as stroke. Thus, participants felt driven to take up expected life roles or duties despite physical impairment or other barriers. For example, female participants persevered in domestic work despite disabilities and male participants were likely to engage in wage-earning activities, such as running a shop, to earn a living.

#### Perceptions of burdening others during participation

Another cultural influence on survivors’ participation was the perception that requiring assistance or accommodation for their disabilities placed a burden on others, which led them to avoid social participation. According to Huang (2011), a central tenet of Chinese culture is collectivism, in which satisfying others is regarded as noble while satisfying oneself is regarded as selfish [45]. This belief has resulted in a widespread expectations of self-reliance, making people feel that they should not depend on others unless it is absolutely required [46], thus manifesting as a significant barrier to stroke survivors’ participation in life situations which often require assistance or involvement from family, friends, or other community members. To address this barrier, it is necessary to help survivors negotiate their cultural perceptions surrounding the idea of requiring help, explain that the feeling of “bothering” others may often be a misconception, and help them understand that their participation is not a burden on others. For example, although loved ones may have to assist survivors in certain aspects of participation, such as pushing their wheelchair or walking at a slower pace, they would be happy to accommodate survivors in these ways as they may welcome their improved mood and active participation in life situations, indicating that the act of helping would bring mutual psychosocial benefits to survivors and those who support them [47].

Based on the study findings, culture may play a significant role in stroke survivors’ reintegration into their communities. While cultural values can pose challenges to social participation, they must also be acknowledged and respected. Although changing values is challenging, we can offer participants alternative perspectives that emphasize the benefits of social engagement for their physical and psychological well-being, thereby motivating them to engage in life activities. Engaging in these activities may be more appealing to stroke survivors if the benefits outweigh cultural concerns. It is also important to emphasize that enhancing their recovery and health can ease the burden on their families. This should encourage stroke survivors to prioritize participation, even if it occasionally burdens others. Conversely, cultural values that promote social participation should be encouraged and leveraged. Tailoring interventions to align with these values is essential, such as suggesting strategies for survivors to return to paid work and handle their household chores more effectively. More favorable outcomes may be attained if interventions take into account these cultural factors.

### Limitations

Some limitations of this study should be considered. First, our findings represent only the views and perspectives of stroke survivors in the study venues in an Eastern Chinese city. Thus, caution should be taken when extending our findings to stroke survivors of other cities or other countries. Second, as we excluded bed-bound stroke survivors or those with moderate or severe cognitive dysfunction or aphasia, the social participation for this group of stroke survivors is not reflected in this study.

### Relevance to practice

This study suggests that routine care in rehabilitation settings should include strategies that promote social participation following a stroke, and the challenges and facilitators should be considered when developing strategies to enhance social participation. Informed by the study findings, for instance, strategies may include helping individuals overcome physical limitations (e.g., rehabilitation exercises, assistive aids recommendations), disseminating stroke-related information, providing psychological encouragement, integrating skills training to enhance communication with family members, and establishing peer support groups in order to strengthen social support networks. Designing tailored interventions also requires particular consideration of cultural values such as the importance of family, filial piety, and societal expectations. To address the cultural-related challenges, appropriate encouragement strategies should be employed, and the interventions should be customized to align with these cultural values.

## Conclusion

Stroke survivors experience different types of participation restrictions in domestic life, community or leisure activities, interpersonal interactions, and returning to work. This study elucidates the impact of cultural values on social participation of stroke survivors, suggesting that cultural beliefs must be factored into the development of interventions aimed at fostering social participation following a stroke. Potential interventions designed to empower stroke survivors may focus on enriching their stroke-related knowledge, offering psychological support, integrating skills training to facilitate better communication with family members, and organizing peer support groups to bolster their social support networks.

## Electronic supplementary material

Below is the link to the electronic supplementary material.

Supplementary Material 1

## Data Availability

The datasets used and/or analysed during the current study are available from the first author on reasonable request.

## References

[1] Feigin VL, Brainin M, Norrving B, Martins S, Sacco RL, Hacke W, et al. World Stroke Organization (WSO): global stroke fact sheet 2022. Int J Stroke. 2022;17(1):18–29. 10.1177/17474930211065917. 34986727 10.1177/17474930211065917

[2] Ytterberg C, Kristensen HK, Tistad Mvon Koch L. Factors related to met needs for rehabilitation 6 years after stroke. PLoS ONE. 2020;15(1):e0227867. 10.1371/journal.pone.0227867. 31940423 10.1371/journal.pone.0227867 PMC6961904

[3] Zawawi NSM, Aziz NA, Fisher R, Ahmad KWalker MF. The unmet needs of stroke survivors and stroke caregivers: a systematic narrative review. J Stroke Cerebrovasc Dis. 2020;29(8):104875. 10.1016/j.jstrokecerebrovasdis.2020.104875. 32689648 10.1016/j.jstrokecerebrovasdis.2020.104875

[4] Kamwesiga J, Bergström A, Bii A, von Koch L, Guidetti S. Experiences of participation in everyday activities for people with stroke in Nairobi, Kenya. Top Stroke Rehabil. 2023;30(5):483–92. 10.1080/10749357.2022.2070360. 35491997 10.1080/10749357.2022.2070360

[5] Rosewilliam S, Roskell CAPandyan A. A systematic review and synthesis of the quantitative and qualitative evidence behind patient-centred goal setting in stroke rehabilitation. Clin Rehabil. 2011;25(6):501–14. 10.1177/0269215510394467. 21441308 10.1177/0269215510394467

[6] Minshall C, Pascoe MC, Thompson DR, Castle DJ, McCabe M, Chau JP, et al. Psychosocial interventions for stroke survivors, carers and survivor-carer dyads: a systematic review and meta-analysis. Top Stroke Rehabil. 2019;26(7):554–64. 10.1080/10749357.2019.1625173. 31258017 10.1080/10749357.2019.1625173

[7] Cheng HY, Chair SY, Chau JP. The effectiveness of psychosocial interventions for stroke family caregivers and stroke survivors: a systematic review and meta-analysis. Patient Educ Couns. 2014;95(1):30–44. 10.1016/j.pec.2014.01.005. 24485756 10.1016/j.pec.2014.01.005

[8] Piskur B, Daniels R, Jongmans MJ, Ketelaar M, Smeets RJ, Norton M, Beurskens AJ. (2014). Participation and social participation: are they distinct concepts? Clin Rehabil.2014; 28(3): 211–220. 10.1177/0269215513499029 10.1177/0269215513499029 23988324

[9] Whiteneck G, Dijkers MP. Difficult to measure constructs: conceptual and methodological issues concerning participation and environmental factors. Arch Phys Med Rehab. 2009;90(11 Suppl):S22–35. 10.1016/j.apmr.2009.06.009. 10.1016/j.apmr.2009.06.009 19892071

[10] Levasseur M, Richard L, Gauvin L, Raymond E. (2010). Inventory and analysis of definitions of social participation found in the aging literature: proposed taxonomy of social activities. Soc Sci Med. 2010; 71(12): 2141–2149. 10.1016/j.socscimed.2010.09.041 10.1016/j.socscimed.2010.09.041 PMC3597625 21044812

[11] World Health Organization. International classification of functioning, disability and health. https://www.who.int/standards/classifications/international-classification-of-functioning-disability-and-health. Accessed 20 May 2024.

[12] Della Vecchia C, Viprey M, Haesebaert J, Termoz A, Giroudon C, Dima A, et al. Contextual determinants of participation after stroke: a systematic review of quantitative and qualitative studies. Disabil Rehabil. 2021;43(13):1786–98. 10.1080/09638288.2019.1679897. 31646906 10.1080/09638288.2019.1679897

[13] de Graaf JA, Schepers VPM, Nijsse B, van Heugten CM, Post MWM, Visser-Meily JMA. The influence of psychological factors and mood on the course of participation up to four years after stroke. Disabil Rehabil. 2022;44(10):1855–62. 10.1080/09638288.2020.1808089. 10.1080/09638288.2020.1808089 32866072

[14] Ribeiro de Souza F, Sales M, Rabelo Laporte L, Melo A, Manoel da Silva Ribeiro N. Body structure/function impairments and activity limitations of post-stroke that predict social participation: a systematic review. Top Stroke Rehabil. 2023;30(6):589–602. 10.1080/10749357.2022.2095086. 10.1080/10749357.2022.2095086 35787246

[15] Mackenbach JP. Cultural values and population health: a quantitative analysis of variations in cultural values, health behaviours and health outcomes among 42 European countries. Health Place. 2014;28:116–32. 10.1016/j.healthplace.2014.04.004. 24835023 10.1016/j.healthplace.2014.04.004

[16] Qiu X, Sit JWH, Koo FK. The influence of Chinese culture on family caregivers of stroke survivors: a qualitative study. J Clin Nurs. 2018;27(1–2):e309–19. 10.1111/jocn.13947. 28677123 10.1111/jocn.13947

[17] Zhang L, Yan T, You L, Gao Y, Li K, Zhang C. Functional activities and social participation after stroke in rural China: a qualitative study of barriers and facilitators. Clin Rehabil. 2018;32(2):273–83. 10.1177/0269215517719486. 28776407 10.1177/0269215517719486

[18] Gu H. Qualitative study on the rehabilitation experience of young and middle-aged stroke patients in the recovery period. Nurs Res. 2019;16:2824–31.

[19] Deng Y, Paul DR. The relationships between depressive symptoms, functional health status, physical activity, and the availability of recreational facilities: a rural-urban comparison in middle-aged and older Chinese adults. Int J Behav Med. 2018;25(3):322–30. 10.1007/s12529-018-9714-3. 29498014 10.1007/s12529-018-9714-3

[20] Leira EC, Hess DC, Torner JC, Adams HP. Rural-urban differences in acute stroke management practices: a modifiable disparity. Arch Neurol. 2008;65(7):887–91. 10.1001/archneur.65.7.887. 18625855 10.1001/archneur.65.7.887

[21] Zimmer Z, Wen M, Kaneda T. A multi-level analysis of urban/rural and socioeconomic differences in functional health status transition among older Chinese. Soc Sci Med. 2010;71(3):559–67. 10.1016/j.socscimed.2010.03.048. 20621749 10.1016/j.socscimed.2010.03.048 PMC2904335

[22] Tong A, Sainsbury P, Craig J. Consolidated criteria for reporting qualitative research (COREQ): a 32-item checklist for interviews and focus groups. Int J Qual Health Care. 2007;19(6):349–57. 10.1093/intqhc/mzm042. 17872937 10.1093/intqhc/mzm042

[23] Francis JJ, Johnston M, Robertson C, Glidewell L, Entwistle V, Eccles MP, et al. What is an adequate sample size? Operationalising data saturation for theory-based interview studies. Psychol Health. 2010;25(10):1229–45. 10.1080/08870440903194015. 20204937 10.1080/08870440903194015

[24] Elo S, Kyngäs H. The qualitative content analysis process. J Adv Nurs. 2008;62(1):107–15. 10.1111/j.1365-2648.2007.04569.x. 18352969 10.1111/j.1365-2648.2007.04569.x

[25] Vaismoradi M, Jones J, Turunen H, Snelgrove S. Theme development in qualitative content analysis and thematic analysis. J Nurs Educ. 2016;6(5):100–10. 10.5430/jnep.v6n5p100.

[26] Lincoln YS, Guba EG, Pilotta J. Naturalistic Inquiry. California: Sage; 1985.

[27] Shenton AK. Strategies for ensuring trustworthiness in qualitative research projects. Educ Inf. 2004;22(2):63–75. 10.3233/EFI-2004-22201.

[28] Korstjens I, Moser A, Series. Practical guidance to qualitative research. Part 4: trustworthiness and publishing. Eur J Gen Pract. 2018;24(1):120–4. 10.1080/13814788.2017.1375092. 29202616 10.1080/13814788.2017.1375092 PMC8816392

[29] Palstam A, Sjödin A, Sunnerhagen KS. Participation and autonomy five years after stroke: a longitudinal observational study. PLoS ONE. 2019;14(7):e0219513. 10.1371/journal.pone.0219513. 31283800 10.1371/journal.pone.0219513 PMC6613678

[30] Cai Y, Towne SD, Bickel CS. Multi-Level factors associated with social participation among stroke survivors: China’s health and retirement longitudinal study (2011–2015). Int J Env Res Pub He. 2019;16(24). 10.3390/ijerph16245121. 10.3390/ijerph16245121 PMC6950688 31847437

[31] Gong S, Sun C, Yang H, Yi X, Hu N, Zhang A, et al. The real experience of stroke patients in young and middle-age adults: a qualitative meta-synthesis. Chin J Nurs. 2021(06 vo 56);843–51. 10.3761/j.issn.0254-1769.2021.06.007

[32] Wang J. Exploring Chinese and Western family culture from a comparative cultural perspective. Science Consulting (Education and Research). 2020; (04):105. https://kns.cnki.net/kcms2/article/abstract?v=IILC1c-FiAEptE1FND0WPRWMJi37YWImY0QV_lxn1IQ5DtPeQj-_BELzYf5LefFzdQRizC__pys7YjwoG1IWIN-Ie5zwm3ZzLcLQIF_YCpVHg16ym2K4Dx2OyZnilYOjiTcWTOqo2AKWi47j3Se7lQ==%26;uniplatform=NZKPT%26;language=CHS

[33] Chen X, He Y, Meng X, Gao C, Liu Z, Zhou L. Perceived participation and its correlates among first-stroke survivors at six months after discharge from a tertiary hospital in China. Arch Phys Med Rehabil. 2018;99(4):667–75. 10.1016/j.apmr.2017.09.120. 29107039 10.1016/j.apmr.2017.09.120

[34] Della Vecchia C, Viprey M, Haesebaert J, Termoz A, Giroudon C, Dima A, et al. Contextual determinants of participation after stroke: a systematic review of quantitative and qualitative studies. Disabil Rehabil. 2019;1–13. 10.1080/09638288.2019.1679897. 10.1080/09638288.2019.1679897 31646906

[35] Nochi M. Loss of self in the narratives of people with traumatic brain injuries: a qualitative analysis. Soc Sci Med. 1998;46(7):869–78. 10.1016/s0277-9536(97)00211-6. 10.1016/s0277-9536(97)00211-6 9541072

[36] Gracey F, Evans JJ, Malley D. Capturing process and outcome in complex rehabilitation interventions: a Y-shaped model. Neuropsychol Rehabil. 2009;19(6):867–90. 10.1080/09602010903027763. 19626556 10.1080/09602010903027763

[37] Wan X, Chau JPC, Mou H, Liu X. Effects of peer support interventions on physical and psychosocial outcomes among stroke survivors: a systematic review and meta-analysis. Int J Nurs Stud. 2021;121:104001. 10.1016/j.ijnurstu.2021.104001. 34246069 10.1016/j.ijnurstu.2021.104001

[38] Cao L, Gao S. Exploration of filial piety and old-age care in traditional Chinese culture. J Shandong Normal Univ (Humanities Social Sci Edition). 2008;0588–91. 10.16456/j.cnki.1001-5973.2008.05.022.

[39] Bassuk SS, Glass TA, Berkman LF. Social disengagement and incident cognitive decline in community-dwelling elderly persons. Ann Intern Med. 1999;131(3):165–73. 10.7326/0003-4819-131-3-199908030-00002. 10428732 10.7326/0003-4819-131-3-199908030-00002

[40] Li N, Gao S. Chinese family concept. J Anyang Normal Univ. 2014;0320–1. 10.16140/j.cnki.1671-5330.2014.03.022.

[41] Xiao S. Intimate power: the intergenerational cooperation and conflicts in childrearing among urban families in contemporary China. J Chin Sociol. 2016;3(1):1–24.

[42] Diprose K, Liu C, Valentine G, Vanderbeck RM, McQuaid K. Caring for the future: climate change and intergenerational responsibility in China and the UK. Geoforum. 2019;105:158–67. 10.1016/j.geoforum.2019.05.019.

[43] Huang B. A brief discussion on the influence of confucianism on Chinese culture. Sci Educ Literature (Second half Month). 2006; (02):126–7.

[44] Zhang X, Pan Y. Advantages of the traditional Chinese family division of labor model. J Jiamusi Educ Coll. 2010; (05):331.

[45] Huang J. Viewing Chinese cultural values and its core collectivism from the perspective of Chinese idioms. Sci Technol Inform. 2011;01535. 10.3969/j.issn.1671-9115.2005.04.014.

[46] Li G. A probe into the origin of Chinese and western family ethics. Stud Ethics. 2005;0466–70. 10.15995/j.cnki.llxyj.2005.04.014.

[47] Yang J. Study on the relationship between satisfying the spiritual and cultural life of the elderly and the culture of filial piety. J Pingyuan Univ. 2004; (04):99–101.

